# Development of a UPLC-MS/MS Method for Simultaneous Determination of Six Flavonoids in Rat Plasma after Administration of *Maydis stigma* Extract and Its Application to a Comparative Pharmacokinetic Study in Normal and Diabetic Rats

**DOI:** 10.3390/molecules22081267

**Published:** 2017-07-29

**Authors:** Bin-Bin Wei, Zai-Xing Chen, Ming-Yan Liu, Min-Jie Wei

**Affiliations:** School of Pharmacy, China Medical University, 77 Puhe Road, Shenyang 110122, China; wbb3127@163.com (B.-B.W.); zaixingchen@sina.com (Z.-X.C.); saffer@163.com (M.-Y.L.)

**Keywords:** *Maydis stigma*, anti-diabetic ingredients, UPLC-MS/MS, rat plasma, pharmacokinetics

## Abstract

*Maydis stigma* is an important medicine herb used in many parts of the world for treatment of diabetes mellitus, which main bioactive ingredients are flavonoids. This paper describes for the first time a study on the comparative pharmacokinetics of six active flavonoid ingredients of *Maydis stigma* in normal and diabetic rats orally administrated with the decoction. Therefore, an efficient and sensitive ultra high performance liquid chromatography coupled to tandem mass spectrometry (UPLC-MS/MS) method for the simultaneous determination of six anti-diabetic ingredients (cynaroside, quercetin, luteolin, isorhamnetin, rutin and formononetin) of *Maydis stigma* in rat plasma has been developed and validated in plasma samples, which showed good linearity over a wide concentration range (*r*^2^ > 0.99), and gave a lower limit of quantification of 1.0 ng·mL^−1^ for the analytes. The intra- and interday assay variability was less than 15% for all analytes. The mean extraction recoveries and matrix effect of analytes and IS from rats plasma were all more than 85.0%. The stability results showed the measured concentration for six analytes at three QC levels deviated within 15.0%. The results indicated that significant differences in the pharmacokinetic parameters of the analytes were observed between the two groups of animals, whereby the absorptions of these analytes in the diabetic group were all significantly higher than those in the normal group, which provides an experimental basis for the role of *Maydis stigma* in anti-diabetic treatment.

## 1. Introduction

Diabetes mellitus (DM) is a metabolic disorder of the endocrine system. It is a global problem that affects the quality of human life. It is quickly becoming the third greatest threat to human health after cancer, cerebrovascular and cardiovascular diseases [1,2]. The prevalence of diabetes is increasing annually, affecting more than 150 million people globally and the projections are pegged at well above 300 million before 2025 [3,4,5]. Diabetes comprises two major types: insulin-dependent diabetes mellitus (type 1 diabetes mellitus) and non-insulin dependent diabetes mellitus (type 2 diabetes mellitus). Over 90% of diabetes cases are type 2 diabetes mellitus [6,7,8]. Therefore, more effective therapies should be urgently developed for the treatment of type 2 diabetes mellitus.

At present, drugs for type 2 diabetes treatment have limitations, such as adverse effects and high secondary failure rates. Healthcare professionals and researchers have explored medicinal herbs with anti-hyperglycemic activities as a complementary treatment [9,10]. *Maydis stigma* (*Zea mays* subsp. *mays* L. [Poaceae]) is the elongated stigmas from the female flowers of maize and it is a waste product of corn cultivation and available in abundance [11,12,13]. It has been used as an aqueous decoction anti-diabetic food drug in folk medicine [14,15,16]. It has great potential, and feasibly a stable commercial product form could be developed to utilize its anti-diabetic effects.

**Figure 1:**
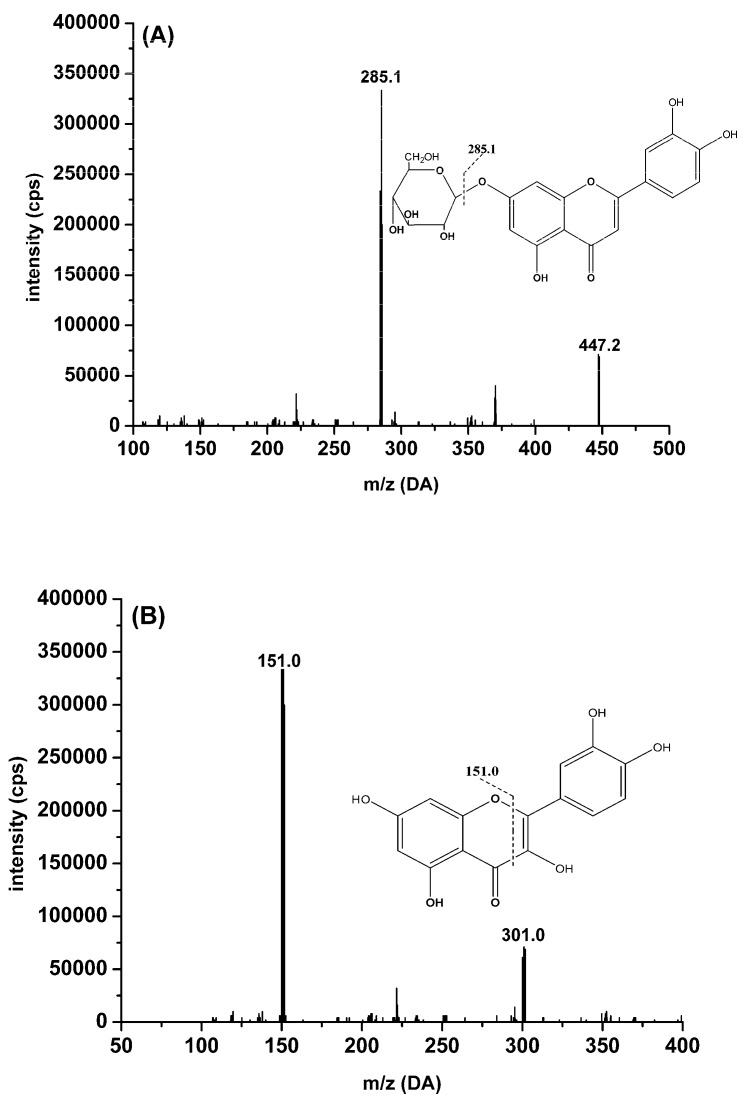

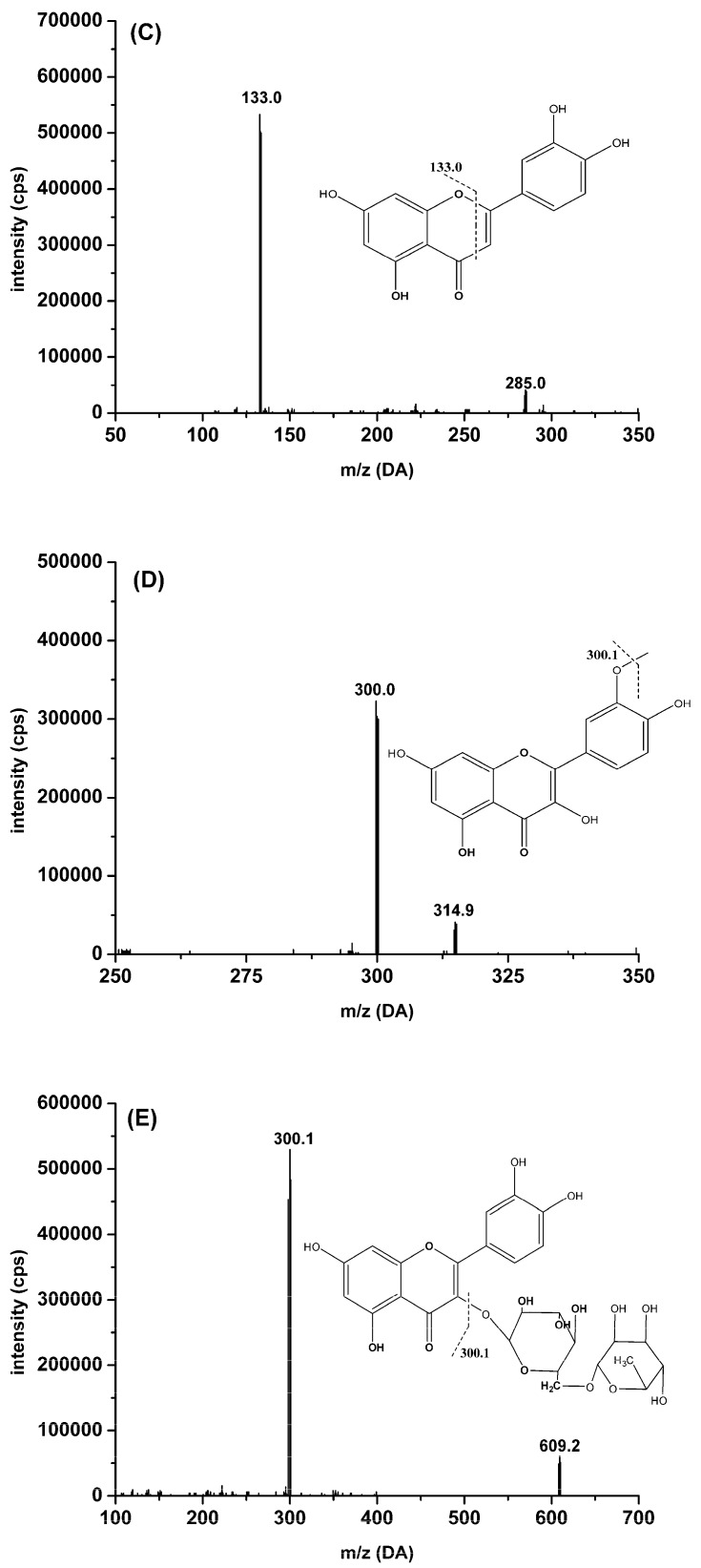

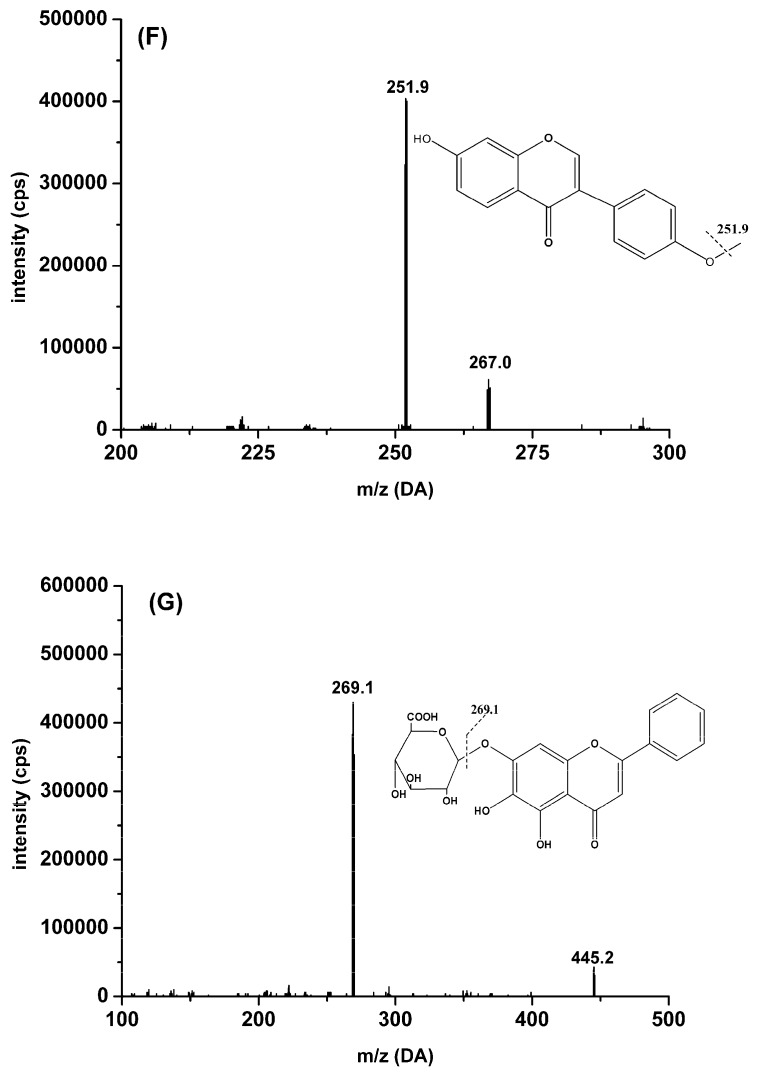
Chemical structures and full scan product ion of precursor ions of cynaroside (**A**), quercetin (**B**), luteolin (**C**), isorhamnetin (**D**), rutin (**E**), formononetin (**F**) and baicalin (**G**; IS).

As previously reported, flavonoids are major active ingredients of *Maydis stigma*, which possess anti-diabetic activities [17,18]. In our experiment, we detected the six flavonoids cnaroside (A), quercetin (B), luteolin (C), isorhamnetin (D), rutin (E) and formononetin (F) (Figure 1). As for the scientific evidence for its anti-diabetic activities, there are a few reports on its pharmacological significance as an oral anti-diabetic and hypoglycemic agent and its effective components, which include phenolic compounds, particularly flavonoids and volatile oils, steroids, alkaloids and saponins [19]. However, there is no detailed report on any LC-MS assay method for the simultaneous determination of these ingredients of *Maydis stigma* for pharmacokinetic investigations. In addition, comparative studies on the pharmacokinetics of these active ingredients between normal and type 2 diabetic rats in plasma after oral administration of *Maydis stigma* decoction provides a new perspective for diabetic treatment.

Therefore, the aim of our study was to develop an efficient, sensitive and selective UPLC-MS/MS method for simultaneous determination of six flavonoid ingredients of *Maydis stigma*, including rutin, cynaroside, luteolin, formononetin, isorhamnetin and quercetin, in rat plasma after oral administration of *Maydis stigma* decoction, and furthermore to reveal any pharmacokinetic differences between normal and type 2 diabetic rats. It was expected that the results of this study can be used to advance the pharmacokinetic and pharmacological studies of *Maydis stigma*.

## 2. Results and Discussion

### 2.1. Optimization of UPLC-MS/MS Conditions

To optimize the proposed UPLC-MS/MS method for simultaneous determination of the six analytes and IS, investigations focused on the effects of several parameters, including proportion of mobile phase, column, and mass-to-charge ratios (*m*/*z*) for parent ions and daughter ions. Effects of these parameters were evaluated and optimized based on peak shape, retention time, sensitivity and signals response.

Negative and positive ionization mode was tested in ESI-MS/MS experiments for optimization of the MS/MS condition. The [M − H]^−^ ions of all the analytes were selected as precursor ions in the MRM transition because they showed higher responses and stabilities than in positive ionization mode for the mass analysis. To obtain chromatograms with good resolution and appropriate retention times, different mobile phases were evaluated. Methanol was found to produce a better response than acetonitrile. With the addition of formic acid to the mobile phase, a significant improvement was observed in the peak symmetry of the analytes. The concentration of formic acid in the water phase was optimized from 0.05% to 0.2%. Finally, 0.1% formic acid in the water phase gave the best peak symmetry, so a methanol −0.1% formic acid in water gradient elution program was used as the mobile phase for the test.

### 2.2. Optimization of Sample Preparation

The protein precipitation (PPT) was used with methanol and acetonitrile, but the recoveries were low and we could not totally eliminate the interferences from the matrix. A liquid-liquid extraction (LLE) method from plasma samples was explored for the six analytes. Different types of solvents and conditions were tested. The results showed that ethyl acetate offered the best recovery compared to extraction with methyl *tert*-butyl ether, ether, or dichloromethane.

### 2.3. Method Validation

#### 2.3.1. Specificity

**Figure 2:**
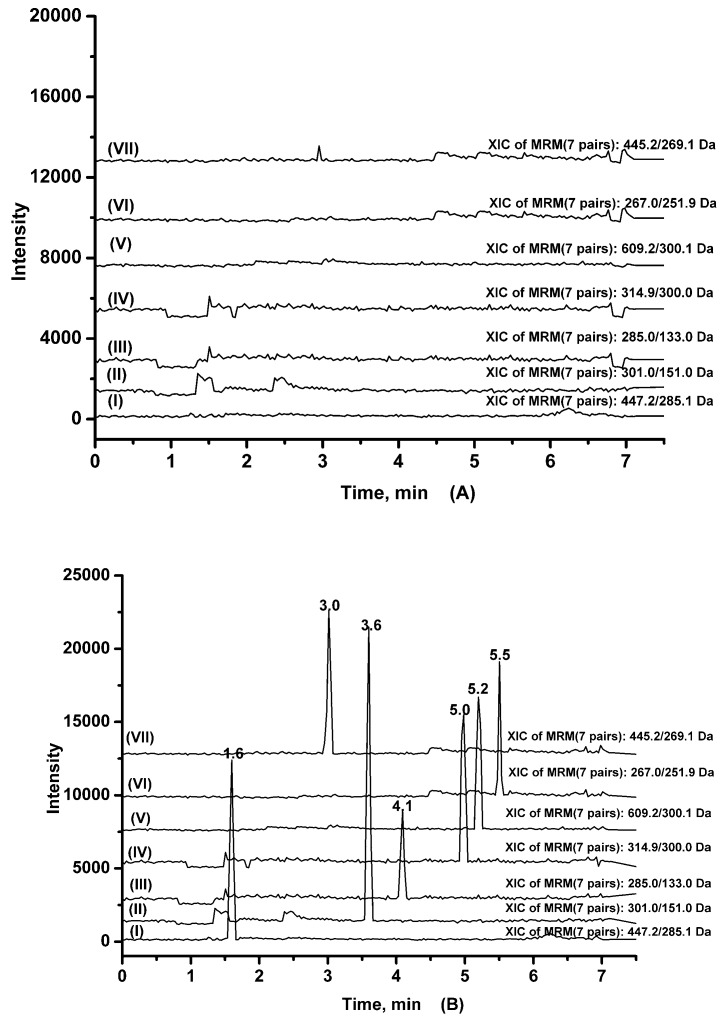

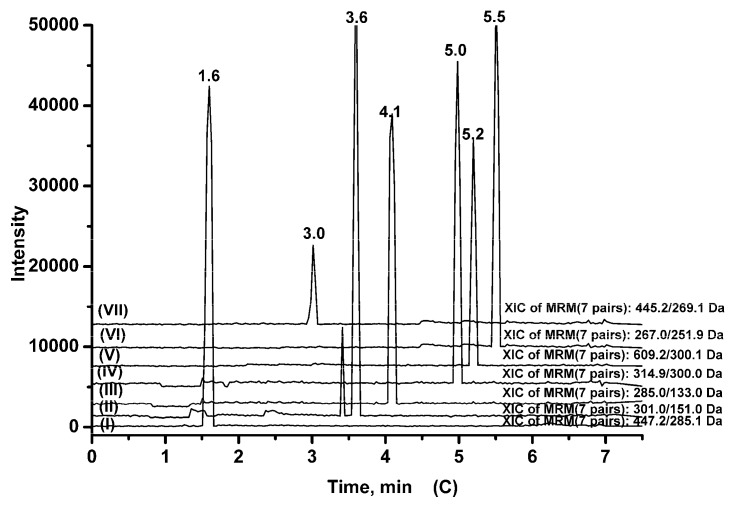
Typical chromatograms of (**A**) blank rat plasma; (**B**) blank rat plasma spiked with six analytes and IS at LLOQ; (**C**) normal group rat plasma sample 2 h after administration of *Maydis stigma* decoction at a dose of 5.0 g·kg^−1^. Representative MRM chromatograms of cynaroside (I), quercetin (II), luteolin (III), isorhamnetin (IV), rutin (V), formononetin (VI) and baicalin (VII; IS).

Method specificity was confirmed by extracting blank rat plasma from six different matrices and comparing the MS/MS responses at the retention times of the analytes. No endogenous interference was observed at retention time of the six analytes and IS. Typical chromatograms of blank rat plasma, blank rat plasma spiked with six analytes and IS at LLOQ, normal group rat plasma sample 2 h after administration of *Maydis stigma* extract at a dose of 5 g·kg^−1^ are shown in Figure 2.

#### 2.3.2. Linearity and LLOQ

The standard calibration curves for spiked rat plasma showed good linearity for the analytes. The results are shown in Table 1. The LLOQ of analytes in plasma were 1.0 ng·mL^−1^.

#### 2.3.3. Precision and Accuracy

The intraday precision, interday precision and accuracy of the six analytes in rat plasma have been validated. All the results of the tested samples were within the acceptable criteria (relative standard deviation (RSD) %: <15%; relative error (RE) %: ±15%). The results are shown in Table 2.

#### 2.3.4. Extraction Recovery and Matrix Effect

The mean extraction recoveries of the six analytes were all more than 85.0%, and the extraction recovery of the IS was 95.2%, the results are shown in Table 2, which indicates the recoveries of the six analytes and IS were consistent and precise in the plasma biosamples. The matrix effect of the six analytes ranged from 85% to 100% at the three QC levels, and the matrix effect of IS was 93.4%, which indicated that there was no significant matrix effect affecting the quantification of the analytes and IS. The results are shown in Table 2.

#### 2.3.5. Stability

Stability studies on the biosamples were run at three QC levels under four different storage conditions. The measured concentration of the analytes deviated within 15.0%. The results are presented in Table 3.

### 2.4. Plasma Pharmacokinetic Study

**Figure 3:**
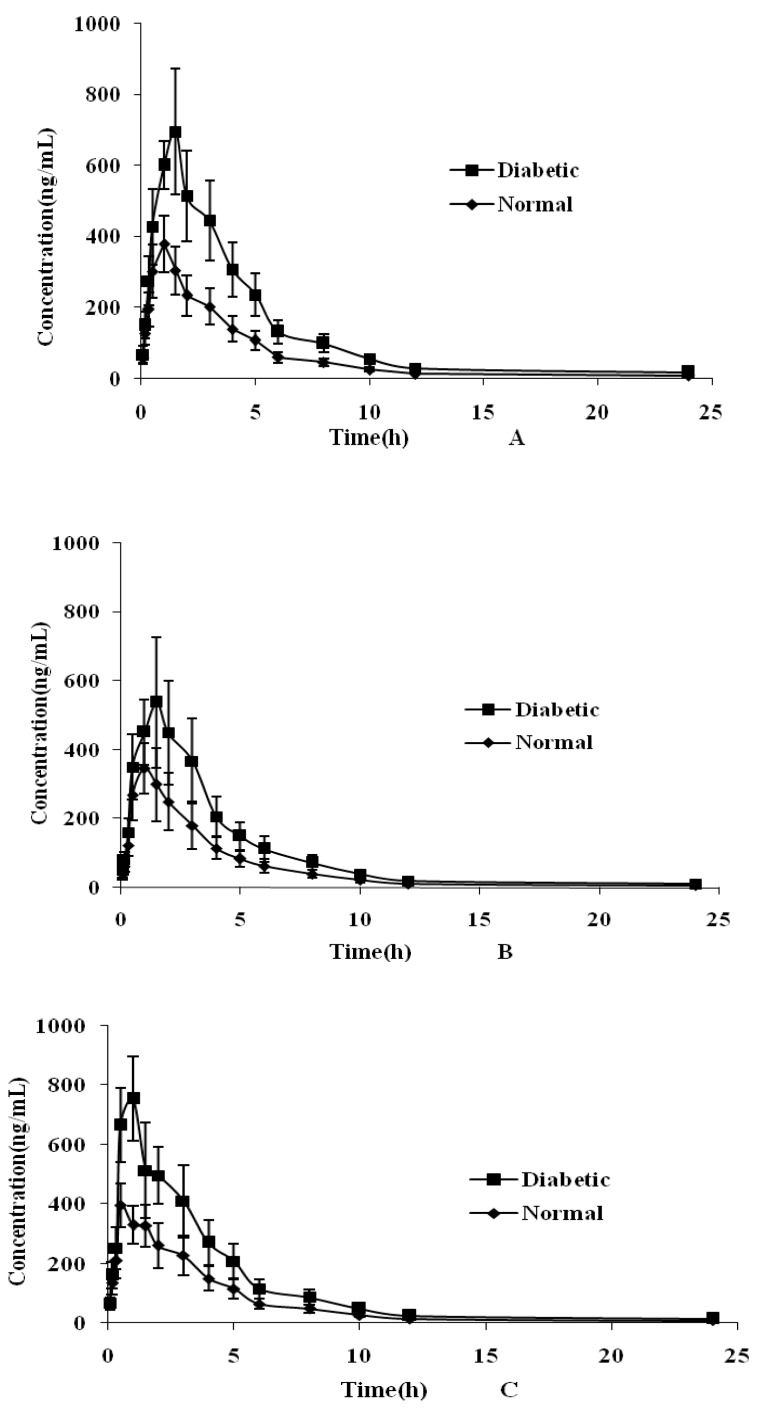

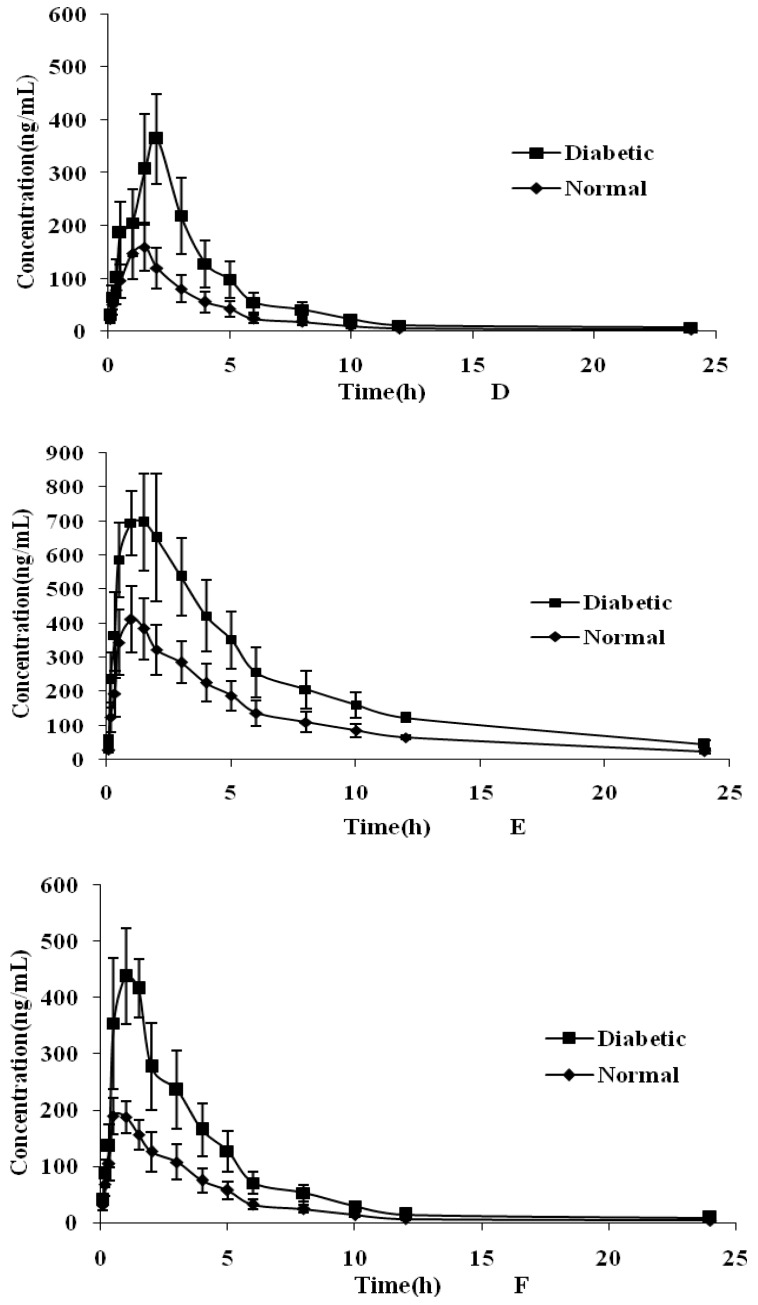
Plasma concentration-time curves for cynaroside (**A**), quercetin (**B**), luteolin (**C**), isorhamnetin (**D**), rutin (**E**), formononetin (**F**) in rat plasma after oral administration of *Maydis stigma* decoction at 5.0 g·kg^−1^ to rats in normal and diabetic groups. Each point represents the mean ± S.D. (*n* = 6).

The utility of UPLC-MS/MS assay for the quantitative analysis of six flavonoids in rat plasma was demonstrated. After oral administration of *Maydis stigma* extract to individual rats (*n* = 6), the mean plasma concentration versus time profiles of six lignans in normal and type 2 diabetic groups are illustrated in Figure 3. The main pharmacokinetic parameters are summarized in Table 4. The results shown that the T_1/2_ (h) of rutin was 8.45 ± 1.06 h, indicating that its metabolic processes in the body were more extended than those of the other analytes. The reason might be attributed to the conversion of quercetin into rutin in the body after a series of changes.

As shown in Figure 3 and Table 4, the results demonstrated that there were significant differences (*p* < 0.05) in pharmacokinetic parameters including AUC_0–t_, AUC_0–∞_, C_max_, T_max_, MRT, and CL_Z_/F between the two groups for the six analytes. A remarkable increase (*p* < 0.05) in the value of AUC_0–t_, AUC_0–∞_, C_max_ and MRT in type 2 diabetic group was noted compared to the normal group. A longer T_max_ was observed for the analytes in the type 2 diabetic group (*p* < 0.05), indicating a slower absorption of the analytes. In contrast, CL obviously decreased (*p* < 0.05) in the diabetic group. The results pointed out that the absorption of the six analytes was increased while the distribution and elimination processes were slowed down in the diabetic group compared to the normal group. The results might be attributed to pathological states of intestinal tract such as delayed gastric emptying and intestinal stasis [20], small intestinal hyperplasia and mucosal hypertrophy that may result in increased absorption of these analytes [21,22]. Type 2 diabetic mellitus can induce several complications such as nephropathy and liver disease [23], and may further affect the metabolism and CL of the analytes. This paper may be useful for deeper studies into the absorption process of *Maydis stigma* in vivo and beneficial for application in preclinical studies.

## 3. Experimental

### 3.1. Materials, Reagents and Animals

*Maydis stigma* were harvested from a maize plantation in the area of Shenbei New District in Shenyang, China and identified at the Department of Traditional Chinese Medicine, China Medical University, Shenyang, China. Rutin, cynaroside, luteolin, formononetin, isorhamnetin and the internal standard baicalin (purity > 98%) were purchased from the National Institute for the Control of Pharmaceutical and Biological Products (Beijing, China). Quercetin (purity > 97%) was separated and purified from *Maydis stigma*, and the structure was validated by comparing the chemical and spectroscopic (UV, NMR and MS) data. Streptozotocin (STZ, purity > 98%) was purchased from Sigma (Saint Louis, MO, USA).

HPLC grade acetonitrile and formic acid were purchased from Fisher Scientific (Fair Lawn, NJ, USA). Ethyl acetate (HPLC grade) was provided by Shandong Yuwang Industrial Co., Ltd. (Yucheng, China).

Male pathogen-free Sprague-Dawley rats (180–200 g) were kindly provided by the Experimental Animal Center of China Medical University. Animal studies were carried out in accordance with the Guideline for Animal Experimentation of China Medical University, and the protocol was approved by the Animal Ethics Committee of the institution.

A diabetic model in rats was induced by the STZ method as described previously [24]. Briefly, the diabetic group animals were intraperitoneally injected with STZ freshly dissolved in a citrate buffer (0.1 mol·L^−1^, pH 4.5) at a single dosage of 45 mg·kg^−1^ body weight. Tail blood glucose value was measured one week later. Rats presenting blood-glucose levels higher than 16.7 mmol·L^−1^ proved the successful implementation of the diabetic model.

### 3.2. Instruments and UPLC-MS/MS Conditions

The controls and samples were analyzed on a 3500 MS/MS system from Applied AB Sciex (Foster City, CA, USA) coupled to an Agilent UPLC 1290 system (Agilent, Santa Clara, CA, USA). Separations were accomplished on an Agilent ZORBAX Eclipse Plus C_18_ (2.1 mm × 100 mm, 1.8 μm) with an Agilent guard cartridge at temperature of 30 °C. The mobile phase consisted of methanol (solvent A) and 0.1% formic acid water (solvent B) which was delivered at a flow rate of 0.3 mL·min^−1^. The linear gradient elution program was as follows: (1) increase from 50% B to 20% B in 0–5 min; (2) hold at 20% B for 1.0 min; (3) from 20% B to 50% B in 0.1 min; (4) hold at 50% B for 1.0 min; the injection volume was 5 μL.

The mass spectrometer was operated in the negative ion mode with a TurboIonSpray source. Table 5 shows the optimized MRM parameters for the analytes and IS. The other ionization parameters were as follows: curtain gas, 20; ion source gas 1, 50; ion source gas 2, 50; source temperature (TEM), 550 °C; entrance potential (EP), 10 V. The dwell time of each MRM transition was 200 ms.

### 3.3. Standard Solution and Quality Control Samples

Standard stock solutions (100 μg·mL^−1^) of the six analytes were prepared by dissolving an appropriate amount of the chemical reference substance in methanol. The stock solutions of the six analytes were further diluted with water/methanol (50:50, *v*/*v*) to obtain standard working solutions. Calibration standard solutions were prepared by spiking a series of mixed working solutions into drug-free rat plasma. These analytes were set at concentrations of 1.0, 5.0, 25, 125, 250, 500 and 1000 ng·mL^−1^. Quality control samples were prepared separately in the 5.0, 250, 800 ng·mL^−1^. Internal standard working solution (100 ng·mL^−1^) was prepared. All calibration standards and QC samples were stored at −20 °C until analysis.

### 3.4. Preparation of Maydis stigma Decoction

The *Maydis stigma* was shade dried to constant weight prior to pulverizing to a fine powder. The dosing solutions used for all animal studies were prepared by the powder (500 g) of *Maydis stigma*, which was extracted with 75% ethanol under reflux for 2 h three times. After concentration under vacuum, the ethanol crude extract was dissolved in 500 mL of water which was the intragastric solution for further use. The decoction was stored in the refrigerator at 4 °C.

### 3.5. Sample Preparation

Plasma samples (100 μL) were transferred to a 10 mL centrifuge tube together with IS solution (10 μL) and water/methanol (10 μL, 50:50, *v*/*v*). After vortex shaking for 30 s, ethyl acetate (1 mL) was added. The analytes and IS were extracted from plasma by vortexing for 5 min and shaking for 5 min. Then the samples were centrifuged at 3000× *g* for 5 min. The organic layer was quantitatively transferred to a 5 mL glass tube and evaporated to dryness at 35 °C under a slight stream of nitrogen. Then, the dried extract was reconstituted in 100 μL solvent (water-methanol, 50:50, *v*/*v*), and 5 μL was injected for UPLC-MS/MS analysis.

### 3.6. Method Validation

The method was fully validated in accordance with US FDA guidelines and ICH [25,26]. The selectivity was tested by compare blank rat plasma with spiked with six analytes and IS at LLOQ. The matrix effect was evaluated by calculating the ratio of the peak area of the analytes spiked into the prepared blank plasma to the peak area in pure standard solutions of the analytes at three QC levels. The acceptable criteria were within ±15%. The linearity of the assay for rat plasma was assessed by analyzing the calibration curves using least-squares linear regression of the peak area ratios of the analytes to the IS versus the nominal concentration of the calibration standard with a weighed factor (1/C^2^). The lower limit of quantification (LLOQ) was defined as the lowest concentration on the calibration curve with an acceptable accuracy within ±20% and the precision below 20%. Mixed QC samples at low, medium and high concentration (5.0, 250, 800 ng·mL^−1^) were analyzed on three separate occasions with six replicates at each concentration per occasion to determine the accuracy and precision. The recovery of the six components were determined at three QC levels with six replicates by comparing the peak areas from extracted samples with those in post-extracted samples spiked with the analytes. The acceptable criteria were within ±15% and the precision below 15%. The stability of biosamples was tested at three QC levels under four different storage conditions: at room temperature for 24 h, at −20 °C for 30 days, after three freeze-thaw cycles and 12 h after prepared at 4 °C.

### 3.7. Method Application

#### 3.7.1. Drug Administration and Sampling

Twelve male rats were divided randomly into two groups with six rats in each. Diabetic model group rats were induced by the STZ method as described previously [24]. The animals were fasted for 12 h with free access to water prior to the oral administration of *Maydis stigma* with a dose of 5 g·kg^−1^ (equivalent to 17.2 mg·kg^−1^ of rutin, 24.5 mg·kg^−1^ of cynaroside, 32.1 mg·kg^−1^ of luteolin, 11.5 mg·kg^−1^ of formononetin, 16.7 mg·kg^−1^ of isorhamnetin and 34.8 mg·kg^−1^ of quercetin). Blood samples (about 250 μL) were collected in heparinized tubes via the postorbital venous plexus veins from each rat before administration and 0.08, 0.17, 0.33, 0.5, 1, 1.5, 2.0, 3.0, 4.0, 5.0, 6.0, 8.0, 10, 12, and 24 h after administration, and were immediately centrifuged and stored at −20 °C until analysis.

#### 3.7.2. Pharmacokinetic Data Analysis

The pharmacokinetic parameters of the six analytes were calculated by the non-compartmental analysis of plasma concentration versus time data using the DAS 2.1 software package (Chinese Pharmacological Society, Shanghai, China). Statistical analysis comparison of area under the curve (AUC), maximum concentration (C_max_), terminal half-life (T_1/2_), time to maximum concentration (T_max_), the sum mean absorption and mean residence time (MRT) and clearance (CL_Z_/F) between two groups were possessed by the SPSS software(version 18.0), while *p* < 0.05 was considered statistically significant for the test. All data were presented as means ± SD.

## 4. Conclusions

In summary, this is the first study to develop a new, efficient and sensitive UPLC-MS/MS method for the simultaneous determination of the six bioactive ingredients of *Maydis stigma* decoction in rat plasma. The method was successfully applied to a comparison of the pharmacokinetic behaviors of the six ingredients in rat plasma decoction between normal and diabetic groups after administration of *Maydis stigma*. The results indicated that the six analytes had a better absorption in diabetic group than the normal group, thus this assay can provide an important basis for better understanding of the in vivo processes of *Maydis stigma* for pharmacokinetic studies. The use of *Maydis stigma* could play an important role in type 2 diabetes mellitus treatment in the future.

## Figures and Tables

**Table 1:** Linear ranges, regression equations and correlation coefficients of multi-components in rat plasma biosamples.

Analytes	Linear Range (ng·mL^−1^)	Regression Equation (×10^−3^)	Correlation Coefficient (*r*)
Cynaroside	1.0–1000	*y* = 3.8*x* + 1.2	0.9946
Quercetin	1.0–1000	*y* = 4.7*x* + 3.1	0.9956
Luteolin	1.0–1000	*y* = 1.3*x* + 2.6	0.9968
Isorhamnetin	1.0–1000	*y* = 2.2*x* + 4.3	0.9976
Rutin	1.0–1000	*y* = 1.8*x* + 6.6	0.9988
Formononetin	1.0–1000	*y* = 3.1*x* + 5.5	0.9967

**Table 2:** Summary of accuracy, precision, recovery and matrix effect of the six analytes in rat plasma (*n* = 6).

Analytes	Concentration (ng·mL^−1^)	Intra-day RSD (%)	Inter-day RSD (%)	Accuracy (RE %)	Recovery (%, mean ± SD)	Matrix Effect (%, mean ± SD)
Cynaroside	5.0	8.2	8.5	8.2	91.2 ± 1.9	93.2 ± 2.4
	125	7.2	5.4	5.8	96.4 ± 4.8	96.2 ± 5.3
	800	5.4	4.1	−4.2	88.5 ± 5.0	94.6 ± 2.8
Quercetin	5.0	9.2	6.7	6.8	85.7 ± 9.3	89.3 ± 7.5
	125	5.9	8.4	5.1	96.3 ± 4.7	91.8 ± 5.0
	800	7.0	4.4	3.0	85.5 ± 3.2	86.2 ± 9.1
Luteolin	5.0	8.7	8.0	5.7	93.6 ± 3.0	92.3 ± 4.6
	125	5.3	2.2	3.1	91.5 ± 2.5	97.8 ± 0.5
	800	6.7	5.3	−2.3	96.9 ± 3.9	89.4 ± 6.3
Isorhamnetin	5.0	9.2	9.8	−5.7	87.1 ± 4.6	91.3 ± 2.3
	125	6.3	7.3	2.2	96.1 ± 8.1	88.3 ± 7.2
	800	5.2	5.8	4.2	93.2 ± 6.3	94.7 ± 6.1
Rutin	5.0	8.8	7.8	−4.7	85.2 ± 4.1	89.1 ± 5.3
	125	5.1	7.5	7.9	92.3 ± 4.4	91.3 ± 6.2
	800	3.4	8.8	5.2	90.3 ± 3.1	94.2 ± 4.3
Formononetin	5.0	9.0	4.9	−7.7	86.2 ± 6.7	92.8 ± 5.7
	125	7.8	5.2	3.9	94.1 ± 3.5	87.0 ± 9.5
	800	6.1	5.8	2.2	96.7 ± 2.7	94.2 ± 3.7

**Table 3:** Stability of the six analytes in rat plasma (*n* = 3).

Analytes	Concentration (ng·mL^−1^)	24 h, Room Temperature		30 Days, −20 °C		3 Freeze-Thaw Cycles		12 h, 4 °C	
		RE (%)	RSD (%)	RE (%)	RSD (%)	RE (%)	RSD (%)	RE (%)	RSD (%)
Cynaroside	5.0	−7.6	10.3	11.2	7.3	10.9	4.1	9.4	5.3
	125	6.8	8.2	5.8	6.2	−6.2	6.3	5.2	8.2
	800	5.3	6.4	4.3	2.9	3.2	7.2	−7.1	6.2
Quercetin	5.0	7.2	4.1	6.4	8.5	−9.6	6.3	8.3	7.2
	125	3.2	3.2	1.2	3.2	−7.3	9.2	5.1	4.3
	800	4.5	5.2	−7.2	6.3	6.4	4.2	8.2	2.9
Luteolin	5.0	−7.4	12.3	13.7	7.8	−4.7	7.0	−3.9	5.9
	125	9.9	5.0	−4.9	3.1	8.8	2.9	1.8	4.9
	800	4.1	4.8	8.1	9.4	9.4	8.3	−7.1	3.8
Isorhamnetin	5.0	−6.7	6.3	8.0	9.1	−11.9	9.3	8.0	13.2
	125	3.4	4.9	−6.5	5.1	−8.9	13.2	6.2	4.0
	800	6.1	5.0	−5.8	8.3	4.8	1.9	7.8	2.2
Rutin	5.0	−9.0	12.1	12.9	8.0	12.2	4.7	10.4	5.1
	125	7.1	10.1	3.2	4.8	−3.5	2.0	3.5	1.2
	800	4.1	7.0	5.5	2.1	−4.7	5.2	−3.7	9.2
Formononetin	5.0	−8.4	13.7	−9.7	8.3	1.9	2.3	−9.3	8.7
	125	−2.7	2.2	−4.4	2.2	−2.7	2.2	−4.7	3.4
	800	2.9	2.2	−2.4	4.2	2.9	2.2	5.0	2.2

**Table 4:** Main pharmacokinetic parameters of the six flavonoids after oral administration of *Maydis stigma* decoction at 5 g·kg^−1^ to rats in type 2 diabetic group, * *p* < 0.05 compared with normal group. (mean ± SD; *n* = 6).

Parameters	Cynaroside	Quercetin	Luteolin	Isorhamnetin	Rutin	Formononetin
Normal group						
AUC_0__–t_ (μgh/L)	1491 ± 341.1	1361 ± 371.3	1620 ± 397.1	608.5 ± 186.3	2688 ± 516.1	792.5 ± 185.8
AUC_0__–∞_ (μgh/L)	1492 ± 341.2	1362 ± 371.5	1621 ± 397.3	608.8 ± 186.5	2944 ± 540.7	793.0 ± 186.2
MRT (h)	4.28 ± 0.15	3.19 ± 0.24	4.01 ± 1.20	4.81 ± 0.22	6.45 ± 0.53	5.23 ± 0.72
T_1/2_ (h)	3.16 ± 0.20	3.07 ± 0.11	5.81 ± 1.16	5.87 ± 1.02	8.45 ± 1.06	5.21 ± 0.74
T_max_ (h)	0.83 ± 0.26	1.05 ± 0.31	0.45 ± 0.25	1.75 ± 0.27	1.08 ± 0.20	0.81 ± 0.21
C_max_ (μg/L)	397.0 ± 78.27	365.8 ± 83.18	414.4 ± 56.58	183.3 ± 33.25	419.5 ± 99.16	204.2 ± 24.31
CL_Z_ (L/kg/h)	17.22 ± 4.251	27.35 ± 8.078	20.79 ± 4.91	29.45 ± 8.090	6.013 ± 1.136	15.13 ± 3.316
Diabetic group						
AUC_0–t_ (μgh/L)	3072 ± 675.7 *	2302 ± 641.5	2876 ± 681.0 *	1370 ± 382.9 *	5017 ± 936.7 *	1745 ± 391.4 *
AUC_0–∞_ (μgh/L)	3074 ± 676.2 *	2303 ± 641.7 *	2878 ± 681.5 *	1371 ± 383.3 *	5498 ± 988.9 *	1746 ± 391.7 *
MRT (h)	4.53 ± 0.15	3.41 ± 0.25	3.82 ± 0.90	5.01 ± 0.42	6.49 ± 0.48	5.53 ± 0.57
T_1/2_ (h)	3.26 ± 0.22	3.21 ± 0.68	5.42 ± 1.03	5.37 ± 0.32	8.65 ± 1.15	5.88 ± 0.54
T_max_ (h)	1.25 ± 0.27 *	1.42 ± 0.20 *	0.63 ± 0.41 *	2.25 ± 0.57 *	1.41 ± 0.49 *	1.25 ± 0.27 *
C_max_ (μg/L)	769.3 ± 111.2 *	551.9 ± 168.3 *	746.2 ± 104.4 *	430.3 ± 40.81 *	744.2 ± 137.1 *	477.1 ± 52.21 *
CL_Z_ (L/kg/h)	8.323 ± 1.966 *	16.21 ± 4.912 *	11.68 ± 2.721 *	12.95 ± 3.402 *	3.216 ± 0.591 *	6.849 ± 1.439 *

AUC_0–t_ AUC_0–∞_, area under the plasma concentration-time curve from time 0 to t, 0 to ∞; MRT, the sum mean absorption and mean residence time; T_max_, time to reach the maximum plasma concentration; C_max_, peak plasma concentration; t_1/2_, terminal elimination half life; Cl, plasma clearance.

**Table 5:** Optimized multiple-reaction-monitoring (MRM) parameters for cynaroside, quercetin, luteolin, isorhamnetin, rutin, formononetin and baicalin (IS).

Analytes	Q1 (amu)	Q3 (amu)	DP (V)	CE (eV)	t_R_ (min)
Cynaroside	447.2	285.1	−70	−35	1.6
Quercetin	301.0	151.0	−70	−33	3.6
Luteolin	285.0	133.0	−70	−40	4.1
Isorhamnetin	314.9	300.0	−70	−33	5.0
Rutin	609.2	300.1	−70	−45	5.2
Formononetin	267.0	251.9	−100	−30	5.5
Baicalin (IS)	445.2	269.1	−70	−35	3.0
